# The Implantable Electrode Co-Deposited with Iron Oxide Nanoparticles and PEDOT:PSS

**DOI:** 10.3390/nano15191511

**Published:** 2025-10-02

**Authors:** Yiyang Liu, Hui Wu, Sheng Wang, Quanwei Yang, Baolin Zhang

**Affiliations:** 1 Collaborative Innovation Center for Exploration of Nonferrous Metal Deposits and Efficient Utilization of Resources, Guilin University of Technology, Guilin 541004, China2120230418@glut.edu.cn (H.W.); 2 Guangxi Key Laboratory of Optical and Electronic Materials and Devices, College of Materials Science and Engineering, Guilin University of Technology, Jian Gan Road 12, Guilin 541004, China; 3 Kedou (Suzhou) Brain–Computer Technology Co., Ltd., Suzhou 215152, China; yangquanwei@kedoubc.com

**Keywords:** iron oxide nanoparticles, implantable neural electrodes, coating, electrophysiological signal transmission, PEDOT:PSS

## Abstract

Iron oxide nanoparticles (IONs) exhibit biocompatibility, ease of drug loading, and potential for generating forces and heat in a magnetic field, enhancing Magnetic Resonance Imaging (MRI). This study proposes coating IONs on electrode surfaces to improve performance and neuron bonding. Methods included synthesizing IONs, grafting chondroitin sulfate (CS), and co-depositing with poly(3,4-ethylenedioxythiophene):poly(styrenesulfonate) (PEDOT:PSS). Results showed reduced impedance, increased charge storage, and improved signal quality in vivo.

## 1. Introduction

Implantable neural electrodes work with electrophysiological acquisition systems to record, analyze, and evaluate the electrical signals generated by neuronal cells [1,2]. They are also widely applied in the treatment of neural system diseases such as epilepsy [3,4] and Parkinson’s disease [5,6]. Electrodes commonly used for implantation include semiconductor-based electrodes, metal wire electrodes, and flexible electrodes of various shapes. Among these, metal wire electrodes are widely employed due to their excellent electrical conductivity combined with high rigidity, which facilitates easier implantation operations [7,8]. During the implantation of electrodes, immune rejection reactions occur [9], which disrupt the local neural environment, leading to the formation of glial scars and inflammation around the electrodes [10]. Currently, the challenge lies in improving the biocompatibility of the implanted electrodes to reduce rejection reactions and increase their function and lifespan [11]. Interface modification between the electrodes and neural tissue is being extensively studied as it can enhance the biocompatibility, stability, signal recording and transmission efficiency of the electrodes while retaining their high mechanical strength and ease of implantation [12,13]. Furthermore, multifunctional interface coatings hold promise in enhancing the electrode’s applicability in specific scenarios. For instance, they can enable the combination of electrophysiological measurement with imaging [14], local drug delivery [15] through the electrode, and impedance measurement for tumor detection [16].

Commonly used materials for electrode modification include platinum [17,18], iridium oxide [19,20], graphene [21,22], various conductive polymers [23,24], etc. These modifiers can, to some extent, reduce the impedance of the electrode and improve its signal capture and conduction function [25], thereby enhancing the communications between tissues and signal analysis systems. However, electrodes still have limitations in applications such as the binding and stimulation of neurons and imaging observation. Here, we use iron oxide nanoparticles (IONs) to modify electrodes, which have the following advantages: (1) IONs are highly biocompatible materials [26]. IONs previously prepared by our group have good water dispersibility and are rich in functional groups such as hydroxyl and amino groups [27]. They can be easily modified or bound by various functional biomolecules, such as hyaluronic acid, chondroitin sulfate [28], streptavidin [29], dimyristoylphosphatidylcholine [30], etc. The modification of chondroitin sulfate has been proven to increase biocompatibility and promote binding with neurons [28]. (2) IONs are able to enhance the image contrast in MRI [31] by shortening the transverse relaxation time (T2) [32,33], so they are often used as contrast agents in MRI. The use of IONs to modify non-magnetic electrodes is expected to improve the image quality of the electrode, fulfilling the aim of observing the position and form of electrode implantation points using MRI. (3) Through the use of IONs as a coating material for implantable electrodes, it is possible to enable drug loading and transport functions of the electrodes [34]. Drugs can alleviate the inflammatory response of the tissue at the electrode implantation interface. This holds potential benefits for therapeutic intervention and tissue compatibility [35]. (4) External magnetic fields can be utilized to apply external forces or heat to the electrodes coated with IONs. This can achieve the investigation of external stimulation effects on neurons [36,37].

PEDOT:PSS is a commonly used material for electrode modification [38,39], which is an ion-electron mixed conductive material [40]. The surface of the electrode modified by PEDOT:PSS forms a nano-porous structure, providing a large surface area, which can reduce electrode impedance and improve conductivity [41]. Incorporating IONs into PEDOT:PSS as electrode modification materials retains the advantages of PEDOT:PSS in improving electrode conduction capacity, and the multifunctionality of IONs has the potential to develop broader applications for electrodes. However, coating IONs on the surface of electrodes is a challenge. In the electrolytic polymerization reaction, the EDOT monomer is oxidized to form cationic radicals, which require the formation of a stable structure with a negatively charged surrounding medium [42]. To incorporate IONs into PEDOT:PSS, it is necessary to make the surface of IONs carry negative charges, which can be achieved by chondroitin sulfate (CS) modification. Additionally, CS is a sulfated glycosaminoglycan with various biological activities [43], and it is widely distributed in the extracellular matrix and cell surfaces. CS has good biocompatibility and low toxicity [44,45], and there are numerous specific CS receptors on the cell membrane. Our previous research has shown that CS modification of IONs can achieve a close integration between neurons [28].

In this work, CS modification was used to modify the surface of IONs, allowing them to participate in the electrolytic polymerization process of EDOT and to deposit on the surface of the implantable electrode. The effects of CS-IONs/PEDOT/PSS coatings on the electrical properties and the ability to record and transmit signals of the implanted electrode were investigated. This work focuses on depositing iron oxide nanoparticles onto electrode surfaces to leverage their exceptional properties for biomedical applications. These applications include targeted drug release and magnetic resonance imaging, while preserving the electrodes’ electrical performance.

## 2. Experiments

### 2.1. Materials

Iron (Ⅲ) acetylacetonate (Fe(acac)_3_, 98%) was purchased from Tokyo Kasei Kogyo Co., Ltd. (Tokyo, Japan). Poly (ethylene glycol) (PEG, Mw = 1000, 95%) was purchased from Xilong Chemistry Co., Ltd. (Shantou, China), chondroitin sulfate (CS, Mw = 499.37, 99%) was purchased from Beijing Solarbio Technology Co., Ltd. (Beijing, China), polyethyleneimine (PEI, Mw = 1800, 99%), 3,4-ethylenedioxythiophene (EDOT, Mw = 142.18, 99%) and Poly (sodium 4-styrenesulfonate) (PSS, average Mw = 70,000, 99%) were purchased from Aladdin Bio-Chem Technology Co., Ltd. (Shanghai, China). Isoflurane (induction at 3–4%, maintenance at 1–2%, flow rate 0.2–0.3 L/min, provided by Shenzhen Ruivode Life Technology Co., Ltd. (Shenzhen, China). sodium pentobarbital (2% concentration, 0.1ml/100g, provided by Zaozhuang Shuitailan Chemical Co, Ltd. (Zaozhuang, China).

### 2.2. Materials and Structure of Electrodes

**Figure 1:**
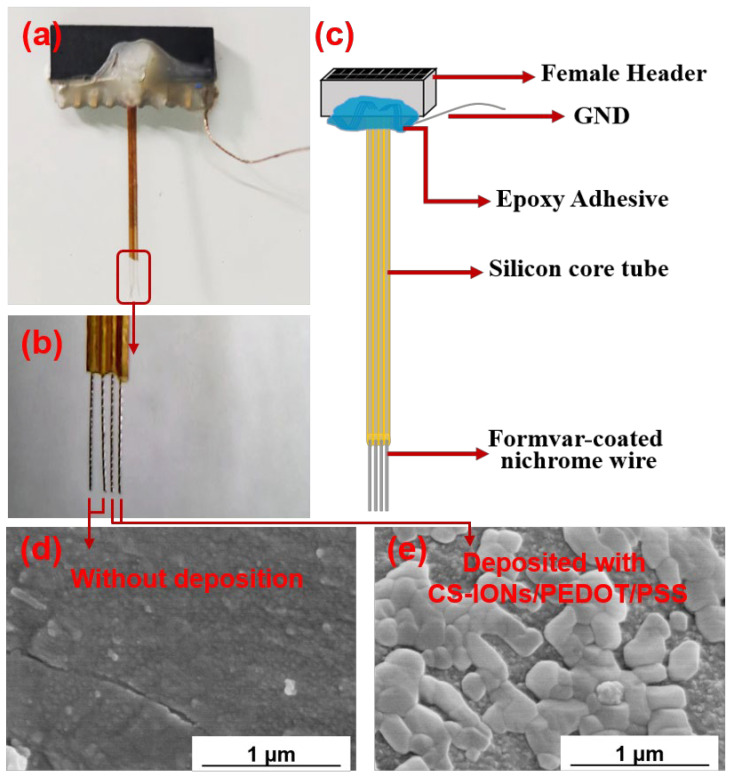
Optical image of the implantable electrode (**a**), magnified image of the nickel-chromium electrodes (**b**), schematic diagram of the implantable electrode material structure (**c**), SEM images of the electrode tips of an electrode without (**d**) and with (**e**) deposition of CS-IONs/PEDOT/PSS.

The nickel-chromium electrodes used in the experiment were produced and provided by the Shenzhen Institute of Advanced Technology, Chinese Academy of Sciences. Figure 1a shows an optical image of an electrode, Figure 1b is the magnified image of the nickel-chromium electrodes of the electrode, and Figure 1c depicts schematically the electrode composition structure. The electrode consists of a female header, ground (GND) wire, silicone tube, and nickel-chromium electrodes. The female header and silicone tube are fixed together with epoxy adhesive. The female header is used to connect to the external electrophysiological signal acquisition system. A single nickel-titanium electrode with a diameter of 25 μm is used throughout its length for contact with the brain. The nickel-chromium electrode is insulated with formvar; the diameter of the end section for contact with the brain is also 25 μm, leaving only the tip exposed as the recording site.

### 2.3. Surface Modification of Iron Oxide Nanoparticles and Deposition of CS-IONs/PEDOT/PSS

The surface charge modification of IONs was carried out to enable their participation as doping materials in the electrochemical polymerization process of EDOT and deposition on the surface of the recording sites of the electrode. The surface charge modification of IONs was achieved through the modification of CS. The specific experimental procedure for synthesizing PEG/PEI/IONs described in previous work [26] is as follows: a CS and IONs mixed solution was prepared with a CS: IONs mass ratio of 2:1 and a CS concentration of 2 mg/mL. The solution was mixed thoroughly and placed in a shaker incubator with reaction parameters of 12 h, 100 rpm, and 20 °C. After incubation, the sample solution was taken out and placed in a dialysis bag with a molecular weight cutoff of 100,000 for 5 days to remove extra CS molecules from the solution. The final product after dialysis is CS-IONs.

Preparation of deposition solution: 5 mL of a 0.6 mg/mL CS-IONs solution was diluted with deionized water to a total weight of 30 g, then PSS powder with a mass fraction of 0.6 wt % (0.18 g) was added to the solution, followed by ultrasonic oscillation for 15 min. Finally, an EDOT solution with a mass fraction of 0.1 wt % (0.03 g) was added, and the mixture was sonicated for 30 min. The solution was stirred and deoxygenated with argon for 10 min before being used for deposition experiments.

Deposition experiments were conducted using an electrochemical workstation with a three-electrode system, employing a constant potential deposition method. A constant potential of 1.0 V was applied between the positive and negative electrodes, and the deposition time was set at 300 s. The electrode is made of nickel-chromium electrodes. Whether a wire was deposited with CS-IONs/PEDOT/PSS was determined by its connection or lack of connection to the external circuit.

The electrochemical impedance spectroscopy (EIS) and cyclic voltammetry (CV) curves of the electrode were tested in phosphate-buffered solution (PBS). The frequency range for the EIS test was 1–100,000 Hz. The voltage range for the CV curve test was −0.6 V to 0.8 V.

### 2.4. Electrode Implantation and Recording of Electrophysiological Signals

Ethics approval and consent to participate in all the animal experiments were obtained in accordance with the National Medical Advisory Committee (NMAC) guidelines and approved by the Guilin University of Technology Experimental Animal Ethics Committee. The approval number is GLUT2024–Y–0870. The process of implanting the electrodes in animals is as follows:

(1) Male Sprague–Dawley (SD) rats, aged 7–8 weeks and weighing 250–300 g, were used in the study. The body weight of each rat was recorded individually prior to experimentation. Animals were housed under specific pathogen–free (SPF) barrier conditions, maintained at 22 ± 2 °C and 50–60% relative humidity, with a 12 h light/dark cycle. They were provided with autoclaved standard rodent diet (crude protein ≥ 18%, crude fat ≥ 4%, crude fibre ≤ 5%; supplied by Beijing Keao Xieli, licence no. SCXK(Beijing)2020-0006) and autoclaved purified water ad libitum, with daily replenishment. All experimental procedures were conducted in accordance with the Guidelines for the Care and Use of Laboratory Animals.

(2) Anesthesia and hair removal: Anesthetize with isoflurane-air supplemented with intraperitoneal injection of a small amount of sodium pentobarbital. Shave the hair on the head of the rat, between the ears, rostral to the eyes, and caudal to the beginning of the neck. The disappearance of the right–angle reflex is used as the criterion for the onset of anesthesia.

(3) Fixation: Secure the rat by attaching an ear bar to the bony depression above the rat’s ear canal, fix the upper incisors with an incisor bar, and adjust the eye socket fixation bar to secure it firmly.

(4) Exposure of the skull and stereotaxic positioning: Disinfect the surgical site with 75% alcohol, iodine, and then 75% alcohol. Cut the skin above the rat’s skull along the midline from the posterior brain to the anterior brain, and use hemostatic forceps to spread the skin. Use small scissors to remove the mucosa on the surface of the skull, and rub the surface of the skull to make it rough for better adhesion of dental cement. Use a stereotaxic instrument for positioning, and select the implantation site at the dDG (Dorsal Dentate Gyrus) brain area of the rat. The mouse remained unconscious throughout the entire process.

(5) Drilling holes and removing the dura mater: Drill 3–5 small holes in the non-implantation area as fixed positions for skull screws, grind the skull at the marked line to expose the skull, and use medical cotton balls moistened with saline to stop bleeding. Use a curved-tip syringe needle to puncture the dura mater. After removing the dura mater, add brain buffer solution to maintain a physiological environment.

(6) Electrode implantation: The electrode wire should be implanted at a 90° angle to the skull plane. Fix the metal micro–wire electrode on the clamp of the stereotaxic instrument, connect it to the input of the signal acquisition system, and ensure that the electrode’s ground reference line is tightly connected to the screw. Mark the depth as 0 when the electrode wire tip touches the surface of the cortex, then slowly lower the electrode wire to the area of signal acquisition.

(7) Noise reduction and signal detection: Before signal acquisition, reduce noise from the system and its surrounding environment to avoid interference from other electrical devices, sound, or physical vibrations.

(8) Dental cement fixation: After cleaning the surface of the skull, use dental cement to fix the electrode. After simple fixation of the electrode, remove the clamp, and seal the skull, screws, and ground wire with dental cement.

Supporting Information shows the image of the electrode implantation device and the signal recording device, respectively.

### 2.5. Characterization

CS-IONs were dispersed in ultrapure water (0.1 mg/mL) and subjected to ultrasonic filtration. The hydrated particle size and zeta potential were measured using a Malvern Nano ZS (Marven City, UK) laser particle size analyzer at 25 °C, with the procedure repeated three times. The dried IONs powder was flattened and scanned by a PANalytical X’Pert PRO (PANalytical, Almelo, The Netherlands) X-ray diffractometer under Cu Kα radiation (40 kV, 30 mA) over a 2θ range of 20–80°. The crystal phases were compared with standard PDF cards. A 2 mg sample was pressed with KBr into a pellet, and infrared spectra were collected in the range of 4000–400 cm^−1^ using a NEXUS 670 Fourier Transform Infrared Spectrometer (Madison, WI, USA) to observe changes in surface functional groups. After being cut and coated with gold, the electrodes were observed using a Hitachi S-4800 scanning electron microscope (manufactured by Hitachi High-Tech Corporation, Tokyo, Japan, with an acceleration voltage of 5–10 kV) to examine surface morphology. Point scanning and area scanning of the target region were performed under SEM, and elemental composition was analyzed using the energy dispersive spectrometer (EDS) attached to the Hitachi S-4800. Cyclic voltammetry was carried out with a three-electrode system in 0.1 M PBS using a CHI-690 electrochemical workstation (Chenhua Instrument Co., Ltd., Shanghai, China). The potential was scanned from −0.6 to +0.8 V at a rate of 0.05 V/s, and the third cycle curve was recorded. Electrochemical impedance spectroscopy was performed with the same workstation and electrode system, over a frequency range of 1 Hz to 100 kHz with an amplitude of 5 mV. The obtained data were fitted using ZView software (Version 3.0.0.22). The electrodes were implanted into the hippocampal region of anesthetized rats. Action potentials and local field potentials (LFP) were recorded using a KD-RHD1 multi-channel electrophysiology acquisition system (Kedou, Yueqing City, China). After filtering, the signal-to-noise ratio and power spectrum were analyzed.

## 3. Results and Discussion

### 3.1. Deposition of CS-IONs/PEDOT/PSS on the Electrode Using the Electrochemistry Method and the Electrodeposition Mechanism

How to coat IONs onto the surface of electrodes is a challenge. In this work, the transformation of the surface charge of IONs from positive to negative was achieved through the modification of CS. The hydrated particle size, zeta potential, FTIR, and XRD of IONs with CS modification were tested. The test results can be found in the Supporting Information Figure S1. The results indicate that CS was successfully modified on the surface of IONs, resulting in the alteration of the surface charge from positive to negative and the types of groups on the IONs, without affecting the phase and crystal structure of the nanoparticles.

**Figure 2:**
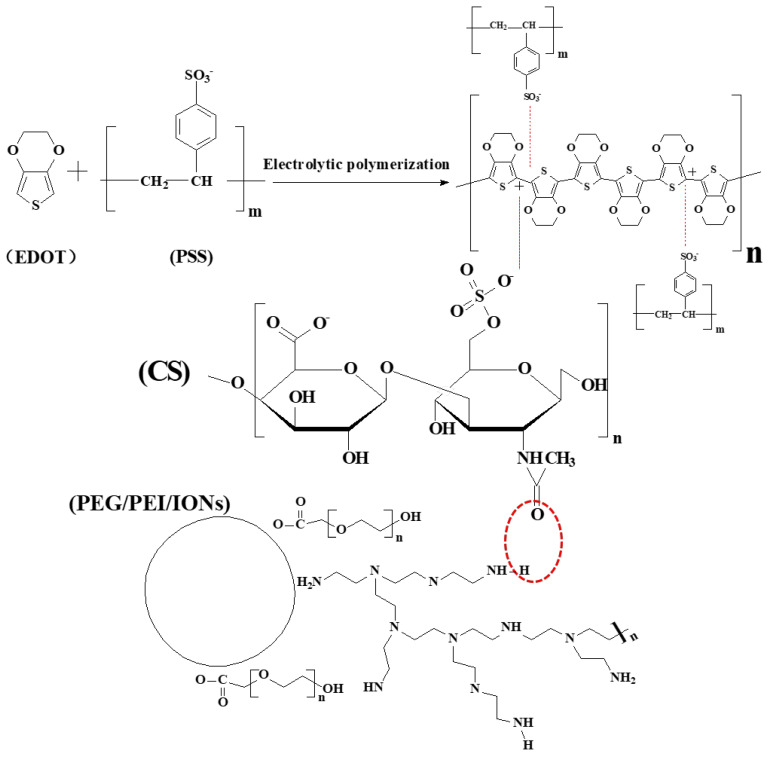
Schematic diagram of the deposition mechanism of IONs: The surface charge of IONs is transformed from positive to negative through CS modification. CS-IONs, along with PSS, participate in the electrochemical polymerization reaction of EDOT and are deposited onto the electrode surface. The red dashed circle indicates the formation of hydrogen bonds between CS and the surface modifiers of IONs.

Consequently, negative surface-charged CS-IONs could participate in the electrochemical polymerization reaction of EDOT along with PSS as dopants, leading to the formation of a CS-IONs/PEDOT/PSS coating on the electrode tip. This method effectively deposits IONs and enables them to tightly bind with the electrode surface. At the electrode-electrolyte interface, the electron conductivity transitions to ion conductivity, making the interface the main site for the electrochemical polymerization reaction. Through the electrochemical polymerization of 3,4-ethylenedioxythiophene monomers, the deposited film encapsulates the electrode tip of the electrode. During the electrolytic polymerization process, PSS acts as a dispersant and dopant due to its hydrophilicity [46], while CS-IONs act as dopants and co–deposit with PEDOT on the electrode tip. In the process of EDOT doping, the EDOT monomer is oxidized to form cationic radicals. These cationic radicals carry energy between the valence band and the conduction band, requiring polarization of the surrounding medium to attain stability [47,48]. Figure 2 shows that the surface of IONs modified by PEG and PEI carries a positive charge, while CS contains a large amount of −O−SO_3_^−^. Using CS to modify IONs enables the conversion of surface charge from positive to negative. The red dashed circle indicates the formation of hydrogen bonds between CS and the surface modifiers of IONs. CS-IONs, along with PSS, act as the dopant and form a stable structure with oxidized EDOT.

### 3.2. Characterization of the Coating and Electrical Performance of the Electrodes

**Figure 3:**
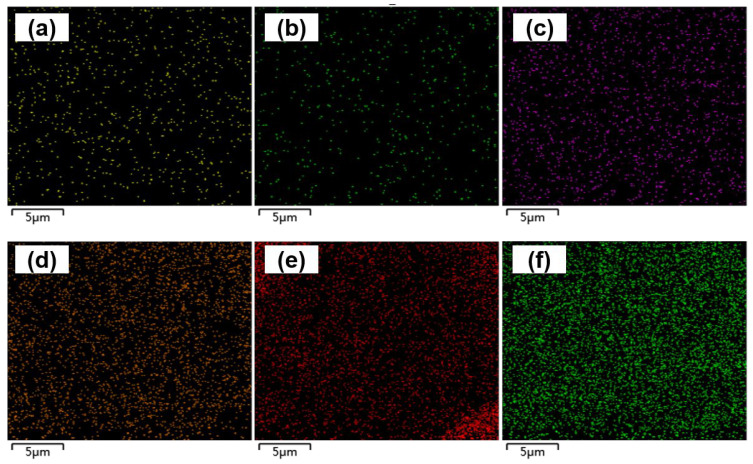
EDS scan images of S element (**a**), C element (**b**), and O element (**c**) on the electrode tip of the electrode without deposition, and S element (**d**), C element (**e**), and O element (**f**) with deposition of CS-IONs/PEDOT/PSS on the electrode tip of the electrode.

Figure 1d,e show SEM images of the surface of the electrode tips at 50,000× magnification without and with the deposition of CS-IONs/PEDOT/PSS material, respectively. It can be seen in Figure 1e that the surface of the electrode tip of the electrode forms a noticeably rough and porous coating material structure after deposition, significantly increasing the effective active area of the electrode tip. EDS surface scans for the primary elements S, C, and O are shown in Figure 3. The changes in the mass and atomic percentages of the electrode surface elements without and with deposition, as analyzed by EDS, are listed in Table 1. The content of Ni and Cr significantly decreases after deposition due to the coating of the deposition film on the nickel-chromium electrode tip of the electrode. Since the materials of the electrode tip of the electrode contain Fe, changes in Fe content are not considered as evaluation criteria. Generally, during the preparation and transportation processes, samples could adsorb low-boiling-point substances or carbon oxides from the air, leading to the detection of C and O even before deposition. However, the significant increase in the content of C and O elements after deposition indicates an increase in the organic components on the modified electrode surface. The additional S element originates from EDOT, PSS, and CS in the deposition film.

**Figure 4:**
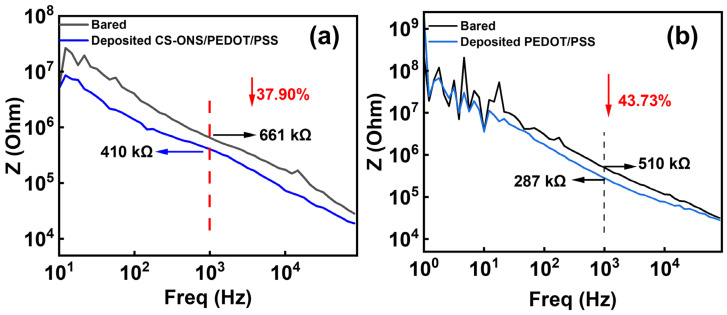
(**a**) Changes in EIS of the electrode electrodes without and with deposition of CS-IONs/PEDOT/PSS. (**b**) Changes in the EIS of the electrode electrodes without and with deposition of PEDOT/PSS.

Figure 4a shows the changes in electrochemical impedance spectra (EIS) at the electrode-electrolyte interface of the electrodes of the electrode with and without being coated with CS-IONs/PEDOT/PSS tested in phosphate-buffer solution (PBS). The frequency of 1000 Hz is chosen for analysis because it is typical for neuronal action potentials [49]. At 1000 Hz, the impedance of the electrode decreases from 661 KΩ to 410 KΩ with a reduction rate of 37.9%. Compared to the bare substrate, the impedance of the PEDOT/PSS—coated substrate decreased from 510 kΩ to 287 kΩ at 1000 Hz, representing an overall reduction of 43.73%. As shown in Figure S2, Groups 1, 2, and 3 indicate the use of deposition solution A containing deionized water, PSS, and EDOT; Groups 4, 5, and 6 indicate deposition solution B, which is deposition solution A supplemented with CS-IONs. Moreover, compared to the bare samples, the impedance of the deposited samples decreased significantly. The impedance reduction was more pronounced for samples deposited with CS-IONs/PEDOT/PSS, resulting in enhanced performance. The electrodes exhibit similar impedance after both deposition processes, indicating comparable capabilities in reducing interfacial resistance and coupling information. The decrease in AC impedance is attributed to the high specific surface area [50] and high ionic conductivity of the coating material. This is supported by the fact that the SEM image shows that the presence of the deposition film significantly increases the specific surface area of the electrode wire tip (Figure 1e), and the PEDOT:PSS components in the deposition film are highly ionically conductive polymers [51].

**Figure 5:**
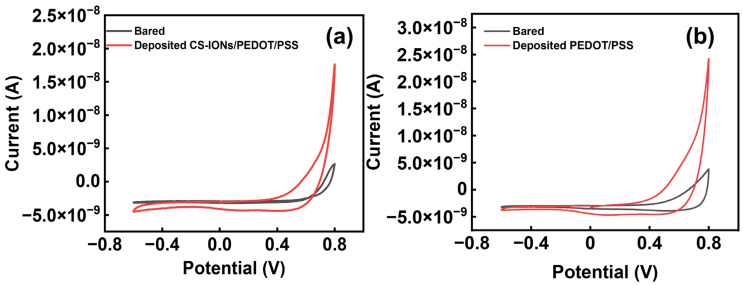
(**a**) The change in one set of CV curve of the electrode electrodes without (bare) and with deposition of CS-IONs/PEDOT/PSS; (**b**) The change in one set of CV curve of the electrode electrodes without (bare) and with deposition of PEDOT/PSS.

During cyclic voltammetry (CV) testing of CS-IONs/PEDOT/PSS and PEDOT/PSS deposits in PBS, the changes in the cyclic voltammetry (CV) curves at the electrode-electrolyte interface for the implantable electrode are shown in Figure 5a,b. After deposition, the area enclosed by the cyclic voltammetry curves increases, corresponding to an increase in the charge capacity density (CCD) [36,52]. The calculation formula for charge quantity is $Q=\frac{1}{V}\int_{E1}^{E2}Id\varphi ,$ E1 and E2, respectively, represent the initial voltage and the final voltage, I represents current, V represents the scan rate, Q represents charge quantity. The calculation formula for charge capacity density is $CCD=\frac{Q}{S}$. The s represents the electrode cross-sectional area. CCD is the charge storage density, which refers to the charge per unit area. Table 2 shows the area of the enclosed region for bared and deposited CS-IONs/PEDOT/PSS electrodes across different groups. After calculating the CCD using the formula, Figure S3 shows that the CCD of the deposited samples significantly outperforms that of the bare electrode, with a substantial increase in charge capacity. Among them, the CV curve of the CS-IONs/PEDOT/PSS deposit exhibits the largest enclosed area, thereby possessing the highest charge capacity and enhancing the capacitive performance of the electrode. Table 3 provides the calculated result of the charge capacity density without and with deposition, as well as the increase ratio. It is observed that the charge density of the electrode increases by 95% after the deposition of CS-IONs/PEDOT/PSS. This indicates that depositing CS-IONs/PEDOT/PSS on the electrode tip enhances the electrode’s charge storage capacity. The increase in electrode charge density indicates a higher amount of charge per unit area after the deposition of the film material on the surface of the implantable electrode, which is analyzed to be due to the higher conductivity of the film material compared to the bulk electrode material.

### 3.3. Evaluation of Neuronal Signal Quality Obtained by the Implanted Electrodes

**Figure 6:**
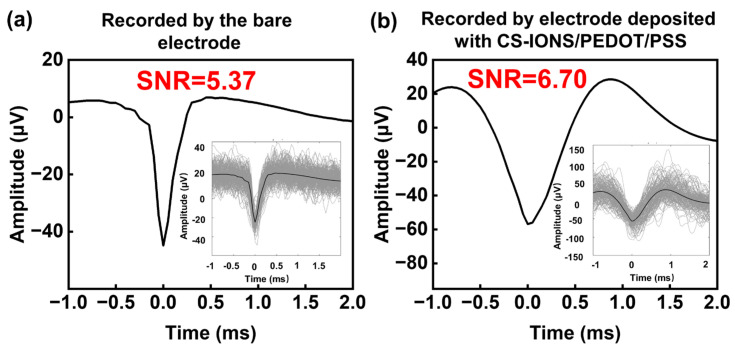
Spike signal recordings of the bare electrode (**a**) and the electrode deposited with CS-IONs/PEDOT/PSS (**b**), showing the raw waveform, average waveform, and signal-to-noise ratio (SNR). Insets display the raw waveform of spike signals.

Figure 6a shows the raw waveform, average waveform, and signal-to-noise ratio (SNR) value of spike signals recorded by the bare electrode of the electrode implanted in the rat brain (Supporting Information Figure S4). The amplitude of spike signals recorded by this electrode is −45 μV, with an SNR of 5.37. Figure 6b presents the raw waveform, average waveform, and SNR value of spike signals recorded by the same electrode but different electrode deposited with CS-IONs/PEDOT/PSS. The amplitude of spike signals recorded by this electrode was −57 μV, with an SNR as high as 6.7. The comparison reveals that the electrode deposited with CS-IONs/PEDOT/PSS recorded spike signals with higher amplitudes and higher SNR values.

**Figure 7:**
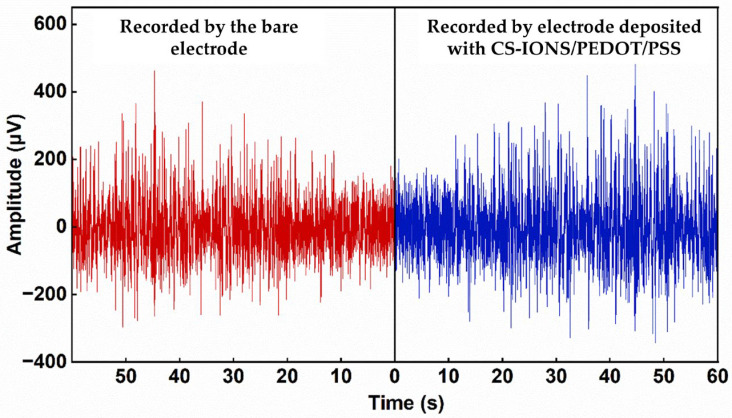
LFP signal waveform recorded by the bare electrode (**left**) and the electrode deposited with CS-IONs/PEDOT/PSS (**right**). The electrode deposited with CS-IONs/PEDOT/PSS exhibits larger amplitudes in the recorded LFP signals.

After 9 days of implantation of the electrode, LFP signals from rat neurons were recorded. Figure 7 compares the LFP waveforms recorded by the bare electrode and the electrode deposited with CS-IONs/PEDOT/PSS. The maximum amplitude of the LFP signal recorded by the electrode coated with CS-IONs/PEDOT/PSS was 549 μV, while that recorded by the bare electrode was only 462 μV. Additionally, we compared electrodes with and without CS-IONs deposition. The LFP signal amplitude on the PEDOT/PSS electrode reached a maximum of 992 μV, significantly exceeding that of the CS-IONs/PEDOT/PSS electrode (Supporting Information Figure S5). Compared to an electrode deposited with PEDOT/PSS, implanting a CS-IONs/PEDOT/PSS deposited electrode into mice resulted in some loss of electrical performance. However, the incorporation of CS-IONs can enhance magnetic properties, providing targeting functionality that is absent in the PEDOT/PSS coating, and increase the effective surface area.

Sampling statistics in Table 4 display the power spectral density (PSD) for the bare electrode and the electrode coated with CS-IONs/PEDOT/PSS at frequencies of 1 Hz, 2 Hz, 4 Hz, 8 Hz, 16 Hz, 32 Hz, 64 Hz, and 128 Hz. PSD represents the power of a signal per unit frequency and is used to describe the distribution of energy across frequencies. The data demonstrate that channels connected with the electrode deposited with CS-IONs/PEDOT/PSS exhibit higher average power, indicating lower signal loss during signal transmission. As shown in Table S1 and Figure S6 (Supplementary Information), the loss of the CS-IONs-coated electrodes was lower than that of bare wires, though higher than that of wires coated with PEDOT/PSS.

In vivo implantation experiments with the electrode indicate that the electrode tip coated with CS-IONs/PEDOT/PSS recorded spike signals and LFP signals with higher quality. We attribute this partly to the functions of CS, which is an important component of the extracellular matrix and is widely distributed around neuronal cell bodies, dendrites, and synapses [53]. Additionally, there are numerous CS-specific receptors on the surface of neuronal cell membranes, allowing CS to bind closely with neurons, assuring excellent biocompatibility [26,54]. This study successfully deposited CS-IONs along with PEDOT:PSS onto the surface of the electrode tip. The CS-IONs/PEDOT/PSS coating enhances the binding ability between the electrode and neuronal cells, which can enhance the recorded LFP signals and neuronal spike signals. This lays the foundation for the development of multifunctional electrodes capable of drug delivery, neural stimulation through heat or force under external electric fields, and enhanced MRI.

## 4. Conclusions

CS-IONs/PEDOT/PSS composite materials were successfully deposited on nickel-chromium electrode tips of the electrode through the electrochemical polymerization of EDOT. The modification of CS converted the positive IONs to negative surface charges, enabling CS-IONs to act as dopants in the electrochemical polymerization process. This deposition significantly reduced electrode impedance and increased charge storage density. Electrode implanted with CS-IONs/PEDOT/PSS in the rat brain recorded neuronal spikes and LFP signals with higher amplitudes, signal-to-noise ratios, and PSD. These improvements are attributed to the CS neuron binding function and the properties of PEDOT:PSS. Further research could explore additional functionalities of these implantable neural electrodes, such as drug delivery, neuron stimulation under a magnetic field, and the enhanced MRI of the implantation site.

## Figures and Tables

**Table 1:** Changes in mass percentage (wt %) and atomic percentage (at %) of surface elements on the electrode tips of the electrode without and with deposition of CS-IONs/PEDOT/PSS.

Element	wt %		at %	
	Bare Electrode Tip	Electrode Tip Deposited with CS-IONs/PEDOT/PSS	Bare Electrode Tip	Electrode Tip Deposited with CS-IONs/PEDOT/PSS
C	7.65	40.13	28.04	60.83
N	0.00	0.00	0.00	0.00
O	0.40	23.59	1.09	26.84
S	0.00	2.58	0.00	1.47
Cr	19.00	10.04	16.10	3.52
Fe	0.82	0.31	0.65	0.10
Ni	72.14	23.34	54.12	7.24

**Table 2:** The area enclosed by the electrodes for different groups: non-deposited (bared) and deposited with CS-IONs/PEDOT/PSS electrodes.

Group Number	Bared Area	Deposited Area
1	1.57897 × 10^−9^	3.7035 × 10^−9^
2	1.6846 × 10^−10^	3.16217 × 10^−9^
3	1.3739 × 10^−10^	5.4120 × 10^−10^
4	1.69545 × 10^−9^	4.06069 × 10^−9^
5	5.4148 × 10^−11^	2.39552 × 10^−9^
6	8.1638 × 10^−11^	4.5103 × 10^−10^

**Table 3:** Analysis of the change in charge capacity density of the electrode without (bare) and with the deposition of CS-IONs/PEDOT/PSS.

Charge Density of the Bare Electrode (c/cm^2^)	Charge Density of the Electrode Deposited with CS-IONs/PEDOT/PSS (c/cm^2^)	Percentage Increase (%)
**1.37 × 10^−3^**	**6.08 × 10^−2^**	**97.95**

**Table 4:** Comparison of PSD at different frequencies between the bare electrode and the electrode with CS-IONs/PEDOT/PSS.

Frequency (Hz)	PSD of the Bare Electrode (dB)	PSD of the Electrode Deposited with CS-IONs/PEDOT/PSS (dB)
1	26.90	27.26
2	24.95	25.52
4	20.87	21.60
8	18.35	19.63
16	13.56	14.94
32	7.85	9.21
64	1.37	2.52
128	−5.17	−4.42

## Data Availability

Data are contained within the article.

## Supplementary Materials

The following supporting information can be downloaded at: https://www.mdpi.com/article/10.3390/nano15191511/s1, Figure S1: The hydrated particle size (a), zeta potential (b), FTIR spectra (c), and XRD patterns (d) of IONs without and with CS modification; Figure S2: Impedance comparison between electrodes with and without IONs/PEDOT/PSS PEDOT/PSS deposition on different groups of electrodes; Figure S3: Comparison of CCD Data for the electrode electrodes without (bare) and with deposition of CS-IONs/PEDOT/PSS and PEDOT/PSS; Figure S4: (a) the image of the electrode implantation device. (b) the image of the signal recording device; Figure S5: LFP signal waveform recorded by the electrode deposited with PEDOT/PSS; Figure S6: Power spectral analysis of LFP signals recorded from channels with PEDOT/PSS, CS-IONs/PEDOT/PSS deposits and channels without any deposits; Table S1: Comparison of PSD at different frequencies between the bare electrode and the electrode deposited with CS-IONs/PEDOT/PSS.
