# Supplementary material for: The Implantable Electrode Co-Deposited with Iron Oxide Nanoparticles and PEDOT:PSS
Source: Nanomaterials (Basel). 2025 Oct 2;15(19):1511. doi: 10.3390/nano15191511 (PMC12525927; doi:10.3390/nano15191511)
Supplement: Supplementary file 1 [file nanomaterials-15-01511-s001.zip › nanomaterials-3794302-supplementary.pdf]

Supporting Information

Article

The Implantable Electrode Co-Deposited with Iron Oxide Nanoparticles and PEDOT:PSS

Yiyang Liu [1,2,3,†], Hui Wu [1,2,3,†], Sheng Wang [1,2,3,*], Quanwei Yang [4] and Baolin Zhang [1,2,3,*]

1 Collaborative Innovation Center for Exploration of Nonferrous Metal Deposits and Efficient Utilization of Resources
2 Key Laboratory of Natural and Biomedical Polymer Materials, Education Department of Guangxi Zhuang Autonomous Region
3 Guangxi Key Laboratory of Optical and Electronic Materials and Devices, College of Materials Science and Engineering, Guilin University of Technology, Jian Gan Road 12, Guilin 541004, China; 1020210185@glut.edu.cn (Y.L.); 2120230418@glut.edu.cn (H.W.)
4 Kedou (Suzhou) Brain–Computer Technology Co., Ltd., Suzhou 215152, China; yangquanwei@kedoubc.com
* Correspondence: wangsheng@glut.edu.cn (S.W.); zhangbaolin@glut.edu.cn or baolinzhang@ymail.com (B.Z.)
† These authors contributed equally to this work

1. The hydrated particle size, zeta potential, FTIR spectra, and XRD patterns of IONs without and with CS modification.

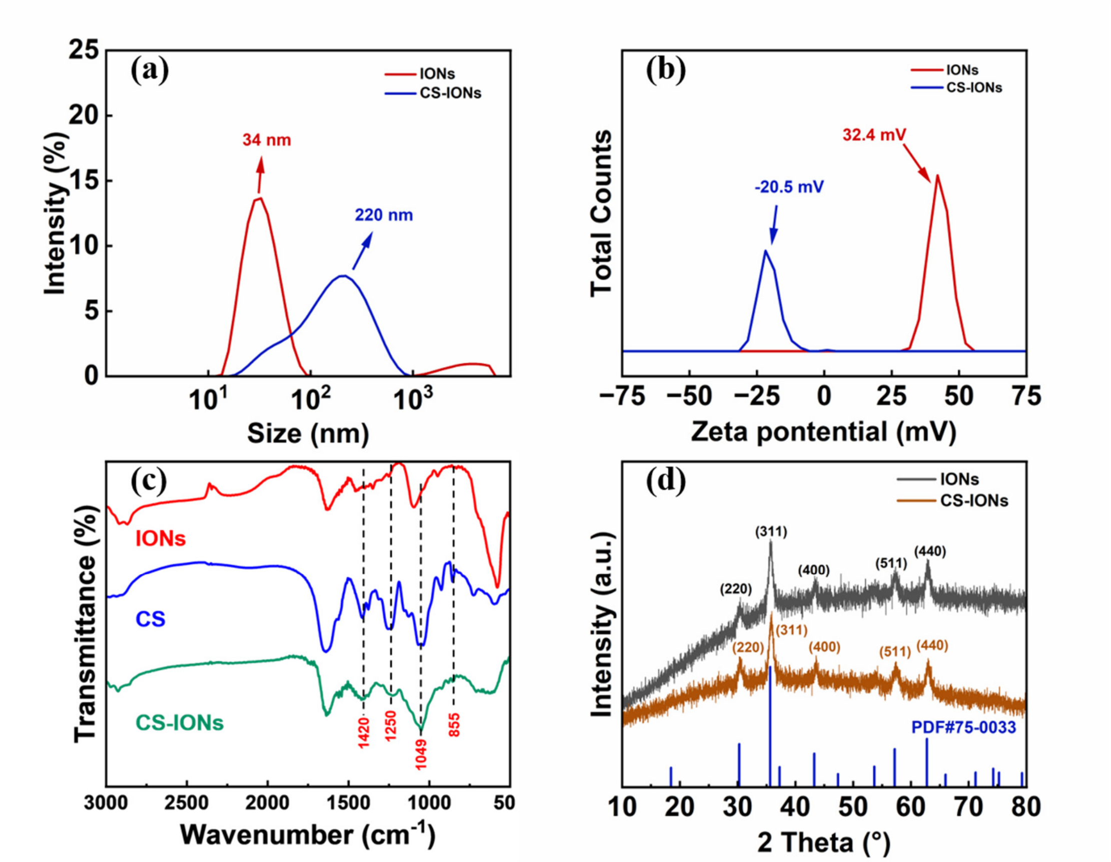

Figure S1. The hydrated particle size (a), zeta potential (b), FTIR spectra (c), and XRD patterns (d) of IONs without and with CS modification.

In Figure S1, after CS modification, the hydrated particle size distribution center increased from 34 nm to 220 nm, and the zeta potential distribution center changed from 32.4 mV to -20.5 mV. The increase in hydrated particle size is attributed to the thickening of the surface modification layer of IONs due to CS modification. The positive charge on the surface of IONs is mainly provided by PEI. After CS modification, the sulfate groups in CS with negative charges provide a large amount of negative charges on the surface of the nanoparticles, causing the zeta potential to change from positive to negative. FTIR spectra show significant changes in the infrared absorption peaks of the nanoparticles after CS modification. The peak at 855 cm^{-1} corresponds to the C-O-S vibration, 1049 cm^{-1} to the C-C vibration, 1250 cm^{-1} to the asymmetric stretching vibration of the S=O bond, and the enhancement of the absorption peak at 1420 cm^{-1} is attributed to the enhancement of the -COOH stretching vibration, which is due to the additional -COOH in CS. The XRD pattern shows no significant changes in the peak position and corresponding peak intensity of the diffraction peaks of IONs without and with CS modification. The diffraction peaks at 2θ values of 30.12°, 35.48°, 43.12°, 57.03°, and 62.63° match those of Fe_3O_4 in the PDF standard card (75-0033), corresponding to the (220), (311), (400), (511), and (440) crystal planes of Fe_3O_4, respectively.

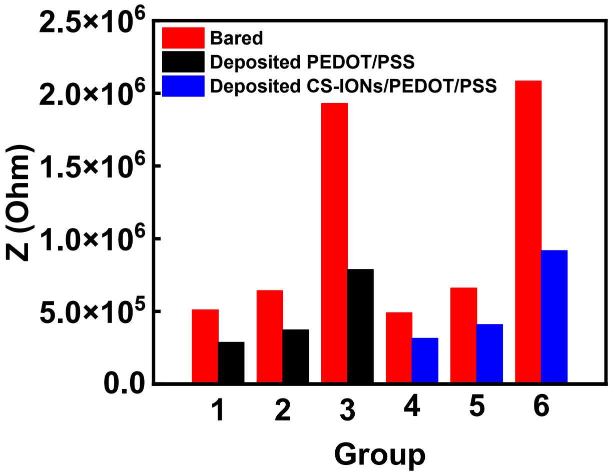

Figure S2. Impedance comparison between electrodes with and without IONs/PEDOT/PSS PEDOT/PSS deposition on different groups of electrodes.

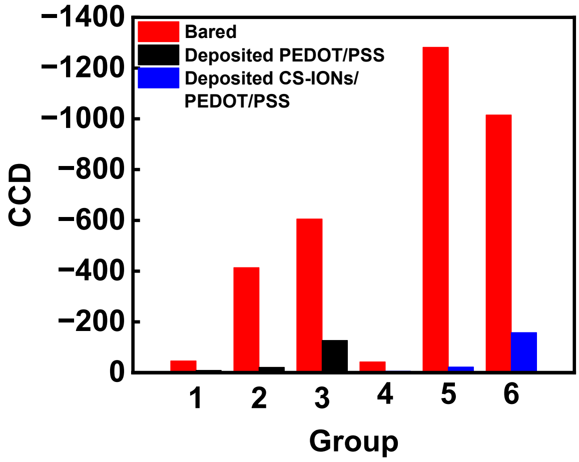

Figure S3. Comparison of CCD Data for the electrode electrodes without (bare) and with deposition of CS-IONs/PEDOT/PSS and PEDOT/PSS.

2. The Process of Electrode Implantation in Animal Experiments

The process of implanting the electrodes in animals is as follows:

(1) Weigh and record the weight of the rats.

(2) Anesthesia and hair removal: briefly anesthetize the rats using isoflurane, followed by intraperitoneal injection of anesthetic. Shave the hair on the head of the rat, between the ears, anteriorly to the eyes, and posteriorly to the beginning of the neck.

(3) Fixation: Secure the rat by attaching an ear bar to the bony depression above the rat's ear canal, fix the upper incisors with an incisor bar, and adjust the eye socket fixation bar to secure it firmly.

(4) Exposure of the skull and stereotaxic positioning: Disinfect the surgical site with 75% alcohol, iodine, and then 75% alcohol. Cut the skin above the rat's skull along the sagittal suture from the posterior brain to the anterior brain, and use hemostatic forceps to spread the skin. Use small scissors to remove the mucosa on the surface of the skull, roughen the skull surface by friction to facilitate the bonding of dental cement. Use a stereotaxic instrument for positioning, and select the implantation site at the dDG brain area of the rat.

(5) Drilling holes and removing the dura mater: Drill 3-5 small holes in the non-implantation area as fixed positions for skull screws, grind the skull at the marked line to expose the skull, and use medical cotton balls moistened with saline to stop bleeding. Use a curved-tip syringe needle to puncture the dura mater. After removing the dura mater, add brain buffer solution to maintain physiological environment.

(6) Electrode implantation: The electrode wire should be implanted at a 90° angle to the skull plane. Fix the metal micro-wire electrode on the clamp of the stereotaxic instrument, connect it to the input of the signal acquisition system, and ensure that the electrode's ground reference line is tightly connected to the screw. Mark the depth as 0 when the electrode wire tip touches the surface of the cortex, then slowly lower the electrode wire to the area of signal acquisition.

(7) Noise reduction and signal detection: Before signal acquisition, reduce noise from the system and its surrounding environment to avoid interference from other electrical devices, sound, or physical vibrations.

(8) Dental cement fixation: After cleaning the surface of the skull, use dental cement to fix the electrode. After simple fixation of the electrode, remove the clamp, and seal the skull, screws, and ground wire with dental cement.

Figure S4(a) and Figure S4(b) show the image of the electrode implantation device and

the signal recording device, respectively.

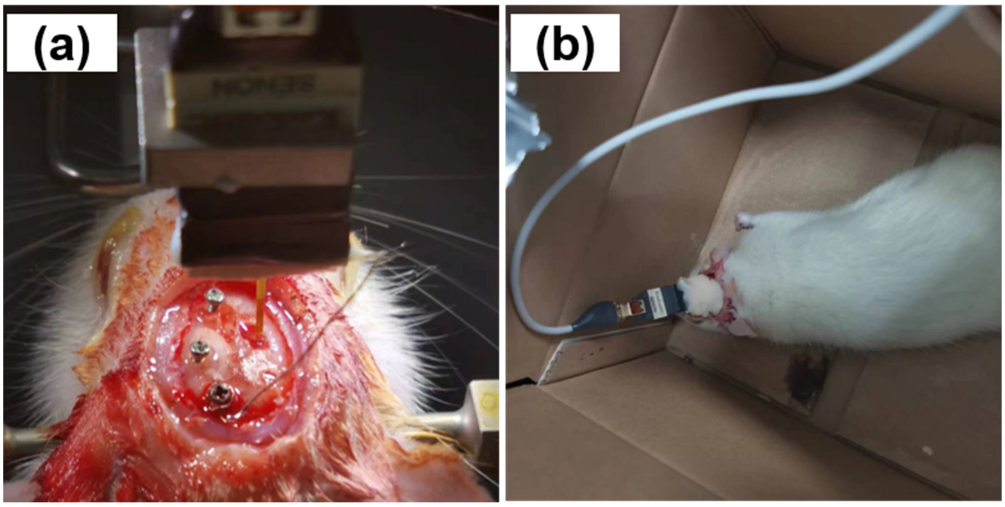

Figure S4. (a) the image of the electrode implantation device. (b) the image of the signal. recording device.

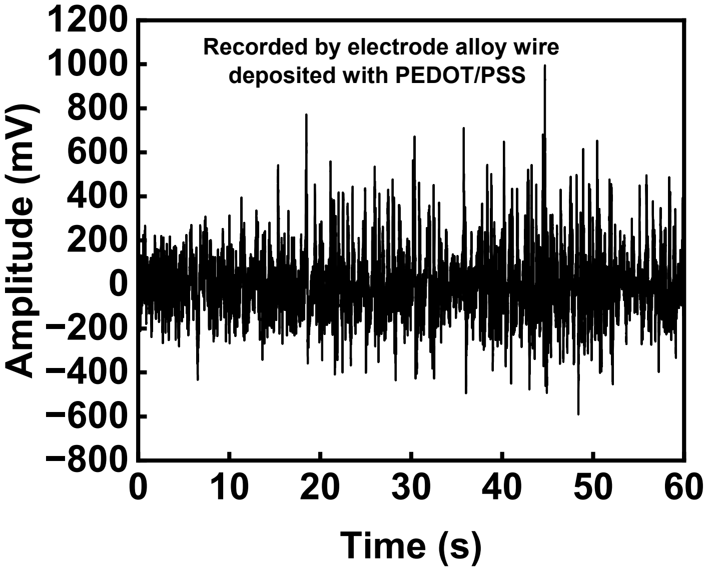

Figure S5. LFP signal waveform recorded by the electrode deposited with PEDOT/PSS.

Table S1. Comparison of PSD at different frequencies between the bare electrode and the electrode deposited with CS-IONs/PEDOT/PSS.

**Frequency (Hz)**	**PSD of the electrode deposited with PEDOT/PSS (dB)**	**PSD of the electrode deposited with CS-IONs/PEDOT/PSS (dB)**

1	32.72	27.26
2	31.12	25.52
4	26.49	21.60
8	24.11	19.63
16	19.09	14.94
32	13.21	9.21
64	6.12	2.52
128	-1.34	-4.42

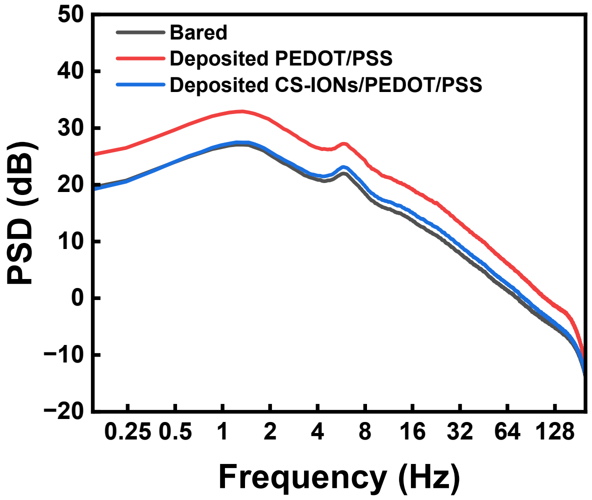

Figure S6. Power spectral analysis of LFP signals recorded from channels with PEDOT/PSS, CS-IONs/PEDOT/PSS deposits and channels without any deposits.
